# Strategy for proactive integrated care for high-risk, high-cost patients/Estrategia de atención proactiva integrada a pacientes en riesgo de alto consumo de recursos

**Published:** 2012-05-29

**Authors:** Xavier Pérez Berruezo, Marc Pérez Oliveras, José María Inoriza Belzulce, Annabel Ibáñez Jiménez, Irene Sánchez Sánchez, Jordi Coderch de Lassaletta

**Affiliations:** Care Management, Baix Empordà Integrated Health Services (SSIBE, Serveis de Salut Integrats Baix Empordà), Palamós, Spain; Primary Care Area, SSIBE, Palamós, Spain; Research group on health services and outcomes (GRESSIRES), SSIBE, Palamós, Spain; Primary Care Area, SSIBE, Palamós, Spain; Primary Care Area, SSIBE, Palamós, Spain; Research group on health services and outcomes (GRESSIRES), SSIBE, Palamós, Spain

**Keywords:** chronic diseases, population morbidity, integrated health care systems, enfermedades crónicas, morbilidad poblacional, prestación integrada de atención de salud

## Introduction

Healthcare models are evolving from healthcare fragmented across centres and levels of care, towards an increasing integration of services. This new model makes it necessary to identify the specific needs of target populations and, based on this, diversify the services provided to better meet the requirements of various population groups and even specific individuals [1].

In recent years, several centres of excellence working in the field of integrated care (Kaiser Permanente, the Veterans Health Administration, and the British NHS, among others) have been dedicating considerable and growing efforts to the identification of the most complex segment of the population by analysing already existing data and the introduction of proactive care models [2, 3].

The Baix Empordà Integrated Health Service (SSIBE *Serveis de Salut Integrats Baix Empordà*) is an integrated health organisation, responsible for the public healthcare services for a catchment population of 125,000. It has a single healthcare structure and also a single electronic medical record database, both of which are clear facilitators for the integration of healthcare [4]. Since 2001, SSIBE has focused a line of research and development on the analysis of morbidity and expenditure considering individuals. We have grouped patients on an ongoing basis by morbidity using Clinical Risk Groups (CRGs) and analysed the relationship of these groups with resource use; it was found that 1% of patients used 22% of the healthcare budget [1, 5].

Given this, we took on the challenge of prioritising healthcare and resources to complex chronic patients. In this context, we developed a predictive model to identify the target population, based on the observed morbidity with high total health expenditure as the dependent variable. This pilot study allowed us to identify factors related to chronicity associated with a greater use of resources [6].

Our objective is to be able to define a proactive healthcare strategy for potentially high-cost patients identified using a predictive model. Once defined, this strategy should allow us to divert resources to and tailor interventions for this type of patient. A second objective is to assess whether the interventions adopted are able to decrease the morbidity compared to the expected rates.

The strategy is divided into two parts: first, we should define the intervention project, the predictive model and its validation. Secondly, we should design a specific intervention that allows us to assess results by performing a clinical trial. The current paper focuses on the first part of the project.

## Methods

The project is being developed in four health areas (HAs), geographical zones for the administration of health in our region, with a total catchment population of 93,233 (16.3% >65 years of age). Two workshops have been held with the key stakeholders involved: primary care doctors, internists, nurses, geriatricians, emergency doctors, and social workers. These workshops were aimed and structured to promote discussion and reach conclusions by consensus. On the basis of these sessions, the interventions to be carried out and corresponding lines of action were established (Table 1).

We built a predictive model for high-cost patients on the basis of age, sex and morbidity according to the CRGs, expenditure on medications >p95, use of hospital drugs in outpatient settings, and hospitalisation. The intervention project was then tested in one of the HAs and its usefulness was assessed.

## Results

These are obtained from three phases:

1. Identification of 4833 high-risk, high-cost patients; 80% being in CRGs 5, 6 and 7 (single dominant or moderate chronic; multiple significant chronic; or three or more dominant chronic conditions). Their previous use of resources compared to other patients is reported in Table 2.
2. Setting up of an alert identifying complex chronic patients in the shared electronic medical records and distribution of these patients by primary care doctor.
3. Specification of the interventions to be carried out and their validation using a pilot, carried out in one of the HAs with the involvement of nine healthcare units and a group of 94 patients. The common actions that should be carried out included reviewing and updating of: prescriptions, and assessment of the level of adherence to medications, attendance to primary care/hospital appointments and more specific issues (such as use of inhalers, dietary control, and social risk). We set aside time in the working day of the professionals for them to carry out these interventions. The programmed interventions included: appointments, home visits and/or telephone consultations. Outcomes can be seen in Table 3.

## Discussion

We set up an overall strategy using a predictive model to identify high-risk, high-cost patients. This identification of such patients facilitates the task of care of primary care doctors/nurses and continuity with the other levels of care. The relatively small number of patients selected and assigned to each primary care doctors/nurses enables personalised analysis and intervention. The intervention defined has been found to be feasible and has made it possible to identify areas of improvement in the monitoring and control of chronic patients by primary care professionals.

In order to act proactively, it is now necessary to define specific interventions. We aim to evaluate the results one year after the introduction of the programme. For this purpose, we plan to carry out a study with the design of a clinical trial to control for the variable “intervention” and quantify its effect.

It is already clear, however, that this strategy must be complemented by other strategies in the areas of the emergency services, hospitalisation and social care, in order to adapt care to meet needs of this type of patient.

##  Conference abstract Spanish

## Introducción

Los modelos de atención sanitaria están evolucionando de una asistencia fragmentada en los centros y niveles, a una integración de servicios. Este nuevo modelo requiere identificar las necesidades específicas de la población asignada y diversificar en consecuencia la oferta de servicios, para adecuarla a las características de los diferentes grupos de población, e incluso individuos concretos [1].

En los últimos años muchas organizaciones excelentes que trabajan en entornos de atención integrada (Kaiser Permanente, Veterans Heath Administration, NHS británico, entre otros) están destinando muchos y crecientes esfuerzos a identificar el segmento de población de mayor complejidad clínica mediante el análisis de información ya existente y la introducción de modelos de atención proactiva [2, 3].

Serveis de Integrats Baix Empordà (SSIBE) es una Organización Sanitaria Integrada, responsable de los servicios sanitarios públicos en una comarca de 125.000 habitantes, de la provincia de Gerona (Cataluña). Dispone de una única estructura asistencial, así como una única historia clínica electrónica, hechos claramente facilitadores para la integración asistencial [4].

Desde el año 2001, SSIBE tiene abierta una línea de investigación y desarrollo sobre el análisis de la morbilidad y el gasto a nivel individual. Categorizamos de forma continuada la morbilidad de nuestra población mediante los Clinical Risk Groups (CRGs), y analizamos su relación con la utilización de recursos. Un 1% de los usuarios consumen el 22% del presupuesto [1, 5].

Nos planteamos el reto de priorizar la atención y los recursos al servicio de los pacientes crónicos complejos. En este marco hemos desarrollado un modelo predictivo para la identificación de la población diana, basado en la morbilidad atendida y en que la variable dependiente es el gasto sanitario total elevado. Esta experiencia nos permite identificar los factores asociados a un mayor consumo de recursos en relación a la cronicidad [6].

Nuestro objetivo es poder definir una estrategia asistencial proactiva sobre pacientes en riesgo de alto coste identificados mediante un modelo predictivo. Esta estrategia definida nos debe permitir orientar los recursos y adecuar las intervenciones a este grupo de pacientes; así mismo evaluar si las intervenciones permiten modificar su morbilidad esperada.

Esta estrategia se divide en dos partes: una primera para definir el proyecto de intervención, el modelo predictivo y su validación, y una segunda para diseñar una intervención que nos posibilite evaluar los resultados mediante un ensayo clínico. El trabajo descrito a continuación se centra en la primera parte del proyecto.

## Metodología

El proyecto se desarrolla en 4 Áreas Básicas de Salud (ABS), con una población de 93.233 personas (16,3% >65 años). Se han realizado dos seminarios de trabajo con los principales agentes implicados: médicos de primaria, internistas, enfermeras, geriatras, médicos de urgencias y asistentes sociales. Los seminarios fueron dirigidos y estructurados para facilitar la discusión y consensuar las conclusiones. De los seminarios se concretaron diferentes ejes de actuación con las intervenciones a realizar (Tabla 1).

Se ha construido un modelo predictivo de pacientes de alto coste en base a edad, sexo, morbilidad según CRG, coste de recetas de farmacia >p95, uso de medicación hospitalaria de dispensación ambulatoria (MHDA) y uso de hospitalización.

Se ha validado el proyecto de intervención en un ABS y se ha valorado su adecuación.

## Resultados

Se distribuyen en tres fases:

1. Identificación de 4.833 pacientes en riesgo de alto coste, el 80% de los cuales pertenecen a los Status de CRG 5-6-7 (“enfermedad crónica dominante única o múltiple”). La utilización previa de recursos que realizan respecto a los otros pacientes se puede ver en la Tabla 2.
2. Implementación en la historia clínica electrónica compartida de una alerta que identifica a los pacientes crónicos complejos. Distribución de los listados de pacientes predichos por médico de primaria.
3. Definición de las intervenciones a realizar y validación de las misma en una prueba piloto, efectuada en 1 ABS con 9 unidades asistenciales y 94 pacientes incluidos. Las acciones comunes realizadas son revisión y actualización de: pauta de medicación, cumplimiento de la prescripción, adherencia a las visitas primaria-hospital, y acciones más específicas (técnicas de inhaladores, control de dieta, valoración del riesgo social). Definimos un espacio en la agenda de trabajo del profesional para llevar a cabo las intervenciones. Se programan diferentes intervenciones: visita, atención domiciliaria y/o atención telefónica. Los resultados se pueden ver en la Tabla 3.

## Discusión

Disponemos de una estrategia global basada en un modelo de predicción del riesgo de pacientes de alto coste. La identificación de los pacientes facilita la intervención del médico/enfermera de primaria y la continuidad de atención con los otros ámbitos asistenciales. El número de pacientes seleccionados y distribuidos por médico/enfermera de primaria hace factible realizar el análisis e intervención personalizada.

La intervención definida ha sido factible y permite identificar aspectos de mejora en el seguimiento y control de los pacientes crónicos por parte de los profesionales de atención primaria.

Es necesaria la definición de intervenciones concretas para actuar de forma proactiva. Se prevé la evaluación de resultados al año de la implementación del programa. Para ello nos planteamos una implementación bajo un diseño de ensayo clínico para controlar la variable intervención y cuantificar su efecto.

Esta estrategia debe ser complementada por otras estrategias en el ámbito de urgencias, hospitalización y sociosanitario que permitan adecuar la atención de estos pacientes.

## Figures and Tables

**Table 1.** Definition of the lines of action for complex chronic patients

Line 1: Identification of the target population
Line 2: Accessibility and coordination of care
Establish a coordinated care pathway for use on discharge to ensure that patient care is well coordinated in primary care
Define and implement emergency care protocol for complex chronic patients
Line 3: Proactive actions for patient monitoring
Line 4: Organisational culture
Make available a guide to social services that defines the procedures to be followed
Create forums to make all healthcare professionals involved aware of the different strategies

**Table 2.** Characteristics of the predicted target population: use of services*

	Predicted cases	Rest of the population	Ratio (A/B)
Acute admissions (episodes)	18.3	3.2	5.8
Total stay in acute hospital (days)	153.0	17.2	8.9
Mean length of stay/admission in hospital (days)	8.4	5.4	1.5
Long-term care/Nursing home admissions (episodes)	6.0	0.2	24.1
Hospital emergency department attendances	6.7	2.8	2.4
Outpatient appointments	473.8	103.6	4.6
Primary care emergency service attendances	13.2	7.2	1.9
Visits to primary care centres	1184.3	356.7	3.3

*Six-month period: data expressed as number per 100 people.

**Table 3.** Outcomes of the pilot project*

Assessed/	Valid cases	% of sample
Prescriptions	87	90.8
Adherence to medication	86	80.2
Compliance with appointments	84	77.4
Proper use of inhalers	45	47.6
Dietary control	82	53.7
Independence (Barthel scale)	77	71.4
Social risk (“*TIRS*” Scale) [7]	33	12.1

*Total n=94 cases.
